# Neuroprotective effects of hesperidin and auraptene on 6-hydroxydopamine-induced neurodegeneration in SH-SY5Y cells

**DOI:** 10.22038/ajp.2024.25214

**Published:** 2025

**Authors:** Malihe Mehrparvar Tajoddini, Elaheh Gheybi, Mehdi Rostami, Seyed Hadi Mousavi, Seyed Isaac Hashemy, Roghayeh Rashidi, Mohammad Soukhtanloo

**Affiliations:** 1 Department of Clinical Biochemistry, Faculty of Medicine, Mashhad University of Medical Sciences, Mashhad, Iran; 2 Medical Toxicology Research Center, Mashhad University of Medical Sciences, Mashhad, Iran; 3 Department of Pharmacology, Faculty of Medicine, Mashhad University of Medical Sciences, Mashhad, Iran; 4 Pharmacological Research Center of Medicinal Plants, Mashhad University of Medical Sciences, Mashhad, Iran

**Keywords:** Hesperidin, Auraptene, ROS, 6-hydroxydopamine, SH-SY5Y cells

## Abstract

**Objective:**

Destruction of dopaminergic neurons causes diseases. Various compounds with neuroprotective and antioxidant properties have been identified, including Hesperidin (HES) and Auraptene (AUR). We aimed in this study to evaluate the *in vitro* protective effects of these compounds in SH-SY5Y neuroblastoma cell line against the induced neurotoxicity of 6-hydroxydopamine (6-OHDA).

**Materials and Methods:**

The MTT test to assess cell viability was used. Flow cytometry was conducted for the cell cycle analysis using propidium iodide (PI) stain. The intracellular production of reactive oxygen species (ROS) was assessed using 2, 7′-dichlorofluorescein diacetate (DCFDA) probe and fluorimetry.

**Results:**

Following 6-OHDA treatment, cell viability decreased, and G2/M arrest and ROS levels increased. Our intervention demonstrated that only HES has neuroprotective effects against 6-OHDA-induced toxicity.

**Conclusion:**

HES protects SH-SY5Y cells against 6-OHDA-induced neural damage via inhibiting G2/M arrest, reducing the amount of ROS, and increasing cell viability. However, the different effects and more precise mechanisms are still unknown, and requires new research on animal and human models.

## Introduction

Parkinson's disease (PD) is caused by the degeneration of nerve cells in a part of the brain called the substantia nigra, with its cause being still unknown and linked to lower dopamine levels (Moore 2003). Resting tremors, postural instability, and gait disturbances result from impaired dopamine secretion and the loss of dopamine-secreting neurons (Kim et al. 2013; Zuo et al. 2017). Researchers do not fully understand the mechanisms underlying neuronal degeneration in PD, but they believe that neuroinflammation, oxidative stress, and excitotoxicity play roles in neuron death (Filho et al. 2016; Rostami et al. 2024). PD, as a debilitating condition, is characterized by the gradual degeneration of dopaminergic neurons which occurs as a result of programmed cell death. This mechanism allows cells to choose their fate in response to excessive damage or adverse conditions (Perier et al. 2012; Venderova and Park 2012). 6-hydroxydopamine (6-OHDA) is commonly employed to provoke oxidative stress and inflict cellular damage in both in vitro and in vivo studies (Haghdoost-Yazdi et al. 2014). Three proposed mechanisms for 6-OHDA's cytotoxicity include 1) auto-oxidation by producing hydrogen peroxide and radicals (Blum et al. 2001); 2) hydrogen peroxide generation via monoamine oxidase (Chiba et al. 1984); and 3) suppression of respiratory chain complex I in mitochondria (Jenner et al. 1992). These factors may either individually or synergistically increase reactive oxygen species (ROS) (Harrison et al. 2005), with heightened cytoplasmic calcium from excitotoxicity or mitochondrial issues leading to cell death (Singh et al. 2010)**.**

Plants, bacteria, and fungi contain more than 1300 phenolic compounds, also known as coumarin derivatives. Citrus fruits, including those from the Rutaceae family such as *Citrus aurantium* and *Aegle marmelos*, contain auraptene (AUR) which is a bioactive monoterpene and coumarin derivative that has been isolated from these sources (Tayarani-Najaran et al. 2021). Previous studies have highlighted AUR's anti-cancer and anti-inflammatory properties (Murakami et al. 2000). Additionally, research indicates that AUR has multiple effects including anti-inflammatory, antioxidant, anti-hypertensive, anti-diabetic, anti-cancer, and neuroprotective properties (Bibak et al. 2019). Another study also showed that AUR can reduce inflammation in the brain that lipopolysaccharide induces (Okuyama et al. 2014). Sweet orange peels and other citrus fruits like tangerines and grapefruits, contain significant amounts of hesperidin (HES), a polyphenolic flavanone glycoside (Crozier et al. 2009; Peterson et al. 2006). Researchers have detected different pharmacological effects of HES such as anti-inflammatory, antioxidant, antihypercholesterolemic, neuroprotective, and anticarcinogenic properties (Abbasinezhad-Moud et al. 2024). Furthermore, HES has shown the ability to protect neurons against various neurodegenerative diseases (Cho 2006)**.**

We aimed in this study to evaluate the protective effects of HES and AUR on the 6-OHDA-induced damage in SH-SY5Y cells.

## Materials and Methods

### Materials

AUR and HES, both with a purity of 97%, were developed by the Gol Elixir Company in Iran. 6-OHDA (purity of ≥ 97%), MTT, RNase A, Triton X-100, propidium iodide (PI), and sodium citrate were procured from Sigma Aldrich (USA). The DMEM/F12 and fetal calf serum (FCS) were provided from Gibco (Life Technologies, NY, USA). The Cellular ROS Assay Kit was also used (Abcam, Cambridge, UK). 

### Cell culture

SH-SY5Y neuroblastoma cell line (Pasteur Institute, Tehran, Iran), which is derived from SK-N-SH cells, is commonly used to assess the neurotoxicity. It is a suitable choice for this investigation due to its extensive prior use in related studies (Kovalevich and Langford 2013; Xicoy et al. 2017). T-25 flasks were used to Cell culture in DMEM/F12 media, supplemented with 10% v/v FCS, and 1% v/v penicillin/streptomycin. Cells were incubated at 37°C with 5% CO_2_ in a humidified incubator.

### Cell viability assay

The MTT assay was used for determining the IC_50_ of 6-OHDA and cytotoxicity of various concentrations of HES and AUR before evaluating their protective effects. Specifically, 1.2×10^4 ^SH-SY5Y cells were seeded in 96-well plates. After 24 h, different concentrations of 6-OHDA (8-1000 µM), HES (8-1000 µM), and AUR (8-1000 µM) (dissolved in 0.08% Dimethylsulfoxide (DMSO) and diluted in DMEM/F12 medium) were applied on the cells, for 24 h. Following treatments, the media containing 0.5 mg/ml of MTT solution was added to each well and incubated for 3 hr. Subsequently, to dissolve the formed formazan crystals, 100 μl of DMSO was applied on each well and the absorbance was assessed at 570 nm using an ELISA reader (Stat Fax 2100, Awareness Technology, USA) (Mousavi et al. 2024).

### Assessment of the HES and AUR protective effects against 6-OHDA cytotoxicity

 In 96-well plates, we seeded 1×10^4^ cells in each well and categorized them to four triplicate groups including control group, HES group treated with 62.5-250 μM of HES for 24 h and exposed to 125 µM 6-OHDA for another 24 h, AUR group treated with 15.5-62.5 μM of AUR under the same conditions, and a 6-OHDA positive control group treated solely with 125 µM 6-OHDA. MTT assay was performed to determination of the cell viability for all groups in triplicate.

### Intracellular ROS assessment

The 2,7′-dichlorofluorescein diacetate (DCFDA) probe was used to measure ROS level (Aranda et al. 2013). Using 96-well plates, 2.5×10^4 ^SH-SY5Y cells were seeded and pre-treated with non-toxic concentrations of HES (62.5-250 μM), and incubated for another 24 h. Cell were washed with 1X buffer from the Cellular ROS Assay Kit, the cells were incubated with 100 μl of 25 μM DCFDA solution in 1X buffer in the dark condition. After re-washing, the cells were treated with 125 µM 6-OHDA, and exposed to the previously specified HES concentrations for 24 h. The control was untreated cells, and N-acetyl cysteine (NAC; 10 mM), known to reduce ROS production, was the negative control. Fluorescence intensity was measured using a Victor X5 Plate Reader (Perkin Elmer, USA) (excitation/emission: 485/535 nm), all samples were tested in triplicate (Memari et al. 2022).

### Cell cycle arrest determination

To evaluate cell cycle arrest and measure dead cells, propidium iodide (PI) and flowcytometry were used (Rashidi et al. 2022). 3×10^5^ cells were seeded in 12-well plates. Pre-treated of the cell was performed using HES (62.5-250 μM), which exhibit protective effects. After another 24 h incubation, 6-OHDA was also added (125 µM), followed by an additional 24 h incubation. After trypsinization, washing, and fixing, the cells were resuspended in PBS, and treated with 200 µl of a solution containing 5 mg/ml of PI, 0.1% Triton X-100, 100 mg/ml RNase A, and 100 μg/ml sodium citrate (Sigma-Aldrich). This suspension was incubated at room temperature in the dark for 15 min. A BD Biosciences flow cytometer was used to analyze DNA content of samples, and the Flow Jo program (version 7.6.1, Tristar, El Segundo, CA) was employed to analyze the cell cycle and the proportions of cells in either the G1, S, or G2/M phases (Tajvar Nasab et al. 2023)**.**

### Statistics analysis

Using GraphPad Prism 8 software (San Diego, CA, USA), one-way ANOVA with Tukey-Kramer post hoc test was performed to evaluate data statistically. Results are shown as mean ± SD, with significance set at p<0.05. Experiments were repeated three times.

## Results

### Non-toxic concentrations of HES and AUR

**Figure 1:**
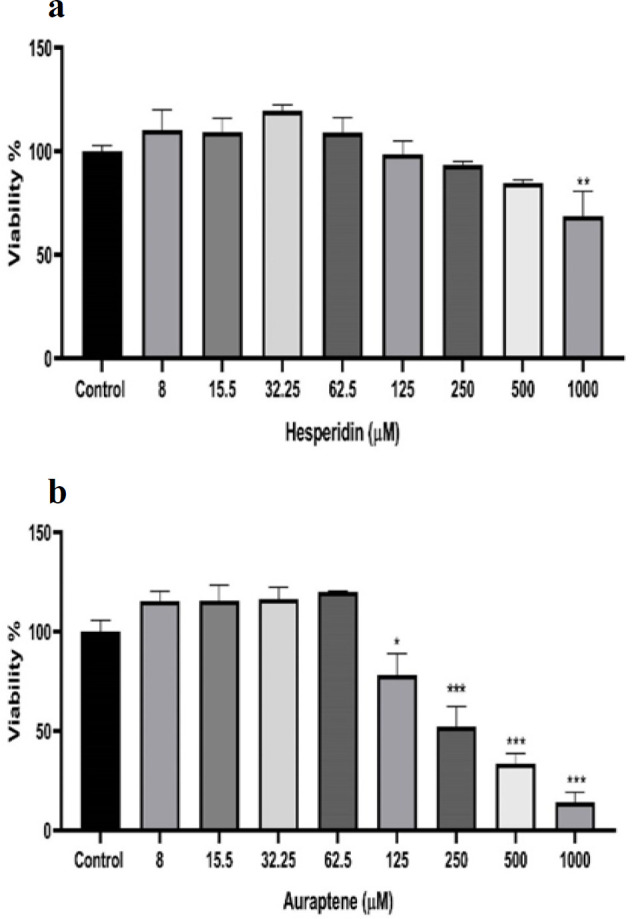
MTT assay results of the effects of HES and AUR on SH-SY5Y cell viability, after a 24 h exposure. (a) HES (8-1000 µM), and (b) AUR (8-1000 µM) are shown. Control cells were untreated. The data was analyzed and is presented as the mean±SD of three independent experiments. Statistical significance is indicated by *p<0.05, **p<0.01, and ***p<0.001 compared to the control group.

Using MTT assay, we evaluated cell viability after treatment with different concentrations of HES and AUR. Results indicated that HES concentrations exceeding 1000 μM and AUR concentrations above 125 μM exhibited cytotoxicity in the cells, as shown in Figures 1a and 1b, respectively. Consequently, the used concentrations were deemed as non-toxic for subsequent experiments.

### 6-OHDA reduced the cell viability

**Figure 2:**
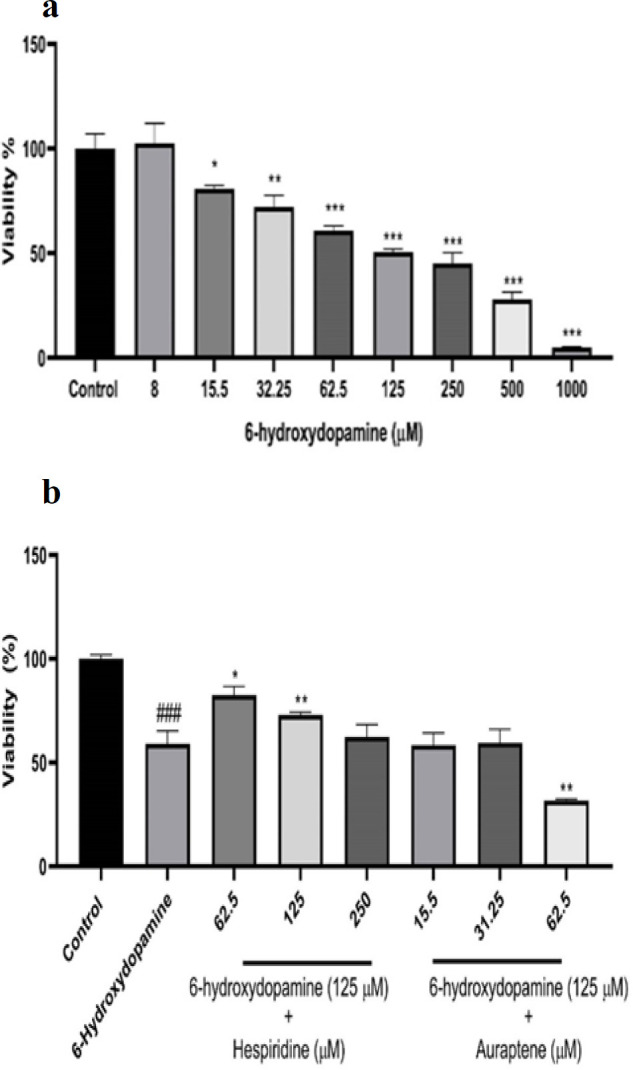
The effect of 6-OHDA on cell viability evaluated by MTT assay after 24 h (a) SH-SY5Y cells were treated with diverse concentrations of 6-OHDA (8-1000 μM). (b) Pre-treatment effects of AUR and HES on the cell viability induced by 6-OHDA. Before exposure to 6-OHDA (125 μM) for 24 h, the SH-SY5Y cells were subjected to AUR (15.5 - 62.5 μM) and HES (62.5-250 μM). The results are the mean±SD of three independent experiments. Statistical analysis revealed *p<0.05, **p<0.01, and ***p<0.001 compared to the 6-OHDA group, whereas ###p<0.001 compared with the control group.

Results indicated that 6-OHDA decreases cell viability in a dose-dependent manner.125 μM of 6-OHDA reduced cell viability by approximately 50% compared to untreated groups, establishing 125 μM as the IC_50_ value for subsequent experiments (Figure 2a).

### Protective effects of HES and AUR against 6-OHDA cytotoxicity

HES pre-treatment (62.5 and 125 μM) significantly improved cell viability. In contrast, pre-treatment with AUR (15.5-62.5 μM) alongside 6-OHDA (125 μM) did not enhance cell viability. AUR at non-toxic levels failed to mitigate 6-OHDA-induced toxicity notably (Figure 2b).

### HES decreased 6-OHDA-induced ROS

**Figure 3:**
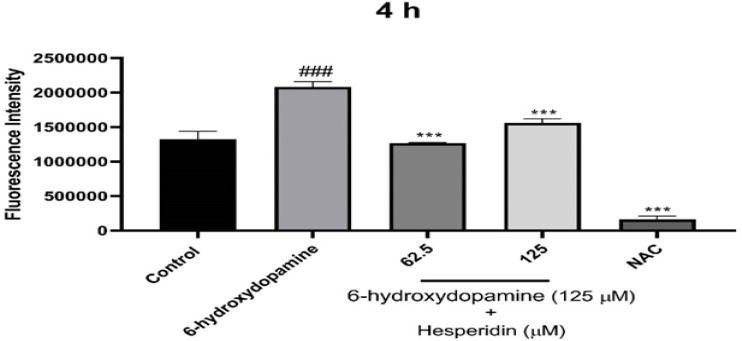
The impact of HES on the ROS production of SH-SY5Y cells induced by 6-OHDA. Before exposure to 6-OHDA (125 μM) for 4 h, SH-SY5Y cells were subjected to HES (62.5 and 125 μM) for 24 h. We present the results as the mean±SD of three independent experiments. Statistical analysis revealed **p<0.01 and ***p<0.001 compared with the 6-OHDA group, ###p<0.001 compared with the control group. N-acetylcysteine (NAC) was used as a negative control.

Based on the ROS results, the cells exposed to 6-OHDA showed significantly higher ROS levels than the untreated control group. However, pre-treatment with HES (62.5 and 125 μM) effectively lowered ROS generation in comparison with the 6-OHDA group. N-acetyl-l-cysteine (NAC) served as a negative control (Figure 3). 

### HES decreases 6-OHDA-induced cell cycle arrest

**Figure 4:**
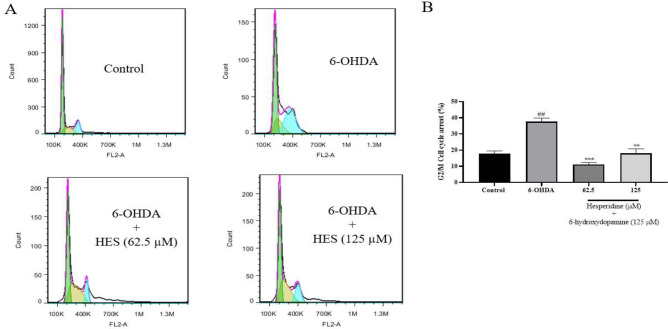
Effects of HES on cell cycle arrest induced by 6-OHDA in SH-SY5Y cells. A flow cytometry histogram (A) of different groups was obtained, along with a column bar graph(B)of the percentage of cells with G2/M cell cycle arrest. We express the data as the mean and standard deviation of three separate experiments. **p<0.01 and ***p<0.001 compared with the 6-OHDA group, ###p<0.001 compared with the control group.

The results of this experiment in SH-SY5Y cells indicated that 6-OHDA caused a significant increase in G2/M cell cycle arrest (39.17 against 16.83 in control, respectively) (p<0.01). However, pre-treatment with HES at 62.5 and 125 μM concentrations significantly decreased 6-OHDA-induced G2/M arrest (10.33 and 16.19, respectively) (Figure 4).

## Discussion

We highlighted the antioxidant and neuroprotective properties of HES, while AUR did not affect the 6-OHDA-induced neurodegeneration in SH-SY5Y cells. We revealed that HES successfully protected the cells from the harmful effects of 6-OHDA toxicity. The underlying mechanism is probably the reduction of cellular ROS, increasing cell viability and inhibition of G2/M cell cycle arrest. Our results showed that AUR could dose-dependently decrease the cell viability. However, AUR was not able to reduce the toxicity exerted by 6-OHDA. 

In contrast, there are several reports describing that AUR can be used as a neuroprotective agent. It has been shown that expression of cyclooxygenase-2 (COX-2) enzyme is inhibited by AUR leading to astrocytes activation, and reduced cell death (Okuyama et al. 2013). In addition, AUR could improve the biochemical and histopathological outcomes in a mouse models of vascular dementia, and enhance learning and memory performance (Mohajeri et al. 2016). Furthermore, in a rat Alzheimer's model, AUR reduced the Bax/Bcl2 ratio in the hippocampus (Joghataee et al. 2020). It has been detected that AUR (30 mg/kg) improved depressive-like behaviors, decreased serum nitric oxide (NO) levels, raised serum malondialdehyde (MDA) levels, and increased serum antioxidant capacity (Amini-Khoei et al. 2022). AUR also exhibited a reduced histopathological change and oxidative stress in the prostate. It inhibited prostate inflammation and demonstrated pro-apoptotic activity (Almukadi et al. 2021). Moreover, AUR (25 mg/kg) reduced brain damage from traumatic injury by lowering MDA, decreasing nitric oxide (NO), reducing oxidative stress, and declining the TNF-α levels as a pro-inflammatory cytokine (Keshavarzi et al. 2021). This could be related to the pro-apoptotic role of AUR reported in some published papers. It has been shown that AUR as a cytotoxic agent causes cell cycle arrest through induction of ROS generation. In contrast to AUR, HES was shown to increase the viability of the cells that were decreased by 6-OHDA. This could be related to the ability of HES to reduce ROS generation and decrease G2/M cell cycle arrest induced by 6-OHDA. HES also exhibited a remarkable dose-dependent ability to inhibit elevation of ROS levels and lipid peroxidation as the indicators of oxidative stress (Chen et al. 2010). A protective role was suggested for HES in histopathological and behavioral changes in 3-nitropropionic acid -induced patients (Menze et al. 2012). Hippocampus histopathological investigations in rat supports the significant reduction of AlCl_3_ toxicity and the preservation of the typical histoarchitecture pattern in these regions with using HES (100 mg/kg) (Justin Thenmozhi et al. 2015).Hesperidin protects against behavioral alterations and loss of dopaminergic neurons in 6-OHDA-lesioned mice (Antunes et al. 2021). Hesperetin decreases the CDKs (cyclin-dependent kinases) and cyclins expression and also increases the expression of p21Cip1 and p27 Kip1 in human breast cancer MCF-7 cells (Choi 2007). It can also change the expression of cell cycle gene through suppressing the MEKK2/MEK5/ERK5 signaling pathway (Deng et al. 2022). 

Overall, we demonstrated that HES effectively reduces oxidative damage induced by 6-OHDA and cell cycle arrest in SH-SY5Y cells. It may be helpful as an adjuvant treatment to reduce oxidative stresses in inflammatory and degenerative brain diseases. Furthermore, it may present potential to be used as a therapeutic compound in further *in vivo* studies to prevent oxidative stress-related brain injury. This warrants further extensive investigation. 

However, the specific effects and mechanisms remain unclear. Our upcoming studies aim to confirm established signaling pathways in animal models and explore the exact mechanisms of HES's action.

 This study evaluated the protective capacity of AUR and HES against induced neurotoxicity by 6-OHDA in the neuroblastoma SH-SY5Y cell line. We showed that HES decreases the harmful effects of 6-OHDA, through decreasing ROS levels and cell cycle arrest in the cells. These findings may propose that oxidative stress and ROS production contribute to 6-OHDA's neurotoxic effects, which are linked to various neurodegenerative diseases. Since HES lowered ROS levels induced by 6-OHDA, it may function as free radical scavenger to protect cells against excitotoxicity in several brain disorders.
